# NMR-driven structure-based drug discovery by unveiling molecular interactions

**DOI:** 10.1038/s42004-025-01542-x

**Published:** 2025-05-31

**Authors:** Gerald Platzer, Moriz Mayer, Darryl B. McConnell, Robert Konrat

**Affiliations:** 1 MAG-LAB GmbH, Karl-Farkas-Gasse 22, 1030 Vienna, Austria; 2 Christian Doppler Laboratory for High-Content Structural Biology and Biotechnology, Department of Structural and Computational Biology, Max Perutz Labs, University of Vienna, Campus Vienna Biocenter 5, 1030 Vienna, Austria; 3 Boehringer Ingelheim RCV GmbH & Co. KG, Dr. Boehringer Gasse 5-11, 1121 Vienna, Austria; 4 Resonate.Bio, Carlbergergasse 66, 1230 Vienna, Austria

**Keywords:** Structure-based drug design, Biophysical chemistry, Solution-state NMR

## Abstract

High-resolution 3D structural information is crucial for drug discovery and routinely used in structure-guided optimization to improve initial hits from screening campaigns to clinical drug candidates. X-ray crystallography is commonly the method of choice to guide medicinal chemistry in the design process, but it has its limitations and shortcomings. Here, we discuss the use of solution-state NMR spectroscopy in combination with selective side-chain labeling and advanced computational workflows to generate protein-ligand ensembles. This provides reliable and accurate structural information about protein-ligand complexes for medicinal chemists that is also suitable for high-throughput.

## Abstract (web summary)

High-resolution 3D structural data are essential for drug discovery, yet X-ray crystallography has limitations in guiding medicinal chemistry. Here, the authors discuss the use of solution-state NMR spectroscopy with selective side-chain labeling and advanced computational workflows to produce accurate protein-ligand ensembles, enhancing structural insights for medicinal chemists and enabling high-throughput applications.

## Introduction

Structure-based drug design (SBDD) has become a cornerstone of modern pharmaceutical research, offering a rational framework for transforming initial hits into optimized drug candidates. By leveraging detailed structural information, SBDD enables the rational design of compounds with enhanced potency and selectivity profiles, ultimately improving the efficiency of the drug discovery pipeline. However, when structural information is unavailable or insufficient, researchers often resort to ligand-based drug design (LBDD), relying on combinatorial chemistry to advance fragments and establish SAR^1^. While effective in certain contexts, LBDD lacks the direct insight into molecular interactions provided by SBDD, underscoring the need for complementary approaches. In the context of increasingly complex biological systems and rising demands for precision therapeutics, SBDD serves as a critical bridge between experimental techniques, computational modeling, and medicinal chemistry.

Molecular recognition of small organic molecules by target proteins relies on numerous non-covalent interactions^2–4^, and therefore the rational and strategic exploitation of these intermolecular interactions to design highly potent and selective binders is a central aim in medicinal chemistry. To date, the required protein 3D structural information is predominantly provided by X-ray crystallography and increasingly by cryo-electron microscopy (cryo-EM). While Cryo-EM represents a powerful alternative method that can generate structures of proteins in various conformational states, the large protein size requirement and lower resolution remain a limitation^5^.

Despite tremendous impact in the past, conventional, purely X-ray crystallography driven structure-based drug discovery suffers from several limitations:

1. Low success-rate of obtaining crystals suitable for structure determination^6^.
  Many proteins do not crystalize readily, a problem that is often associated with the inherent flexibility of certain proteins, or the existence of flexible linker regions that connect different sub-domains of a full-length transcript. Additionally, the presence of post-translational modifications can hinder crystallization by introducing heterogeneity in the structural ensemble.
2. High-throughput soaking systems are difficult to establish.
  Soaking small molecules into pre-formed protein crystals is often complicated by poor solubility or aggregation of the compounds, which can prevent proper diffusion into the crystal. Additionally, some ligands may destabilize or damage the crystal lattice, causing degradation of crystal quality. High-throughput soaking systems also face challenges in maintaining consistent crystal size and quality, and certain binding events may be too weak or transient to be captured by soaking. Furthermore, pre-formed crystals may trap the protein in a conformation that is not conducive to optimal ligand binding, limiting the ability to screen ligands efficiently.
3. Molecular interactions are inferred and not physically measured.
  In X-ray crystallography, the electron density maps generated provide an indirect representation of molecular structures, where interactions between ligands and proteins are inferred from the spatial arrangement of atoms, rather than being directly observed. This means that key binding interactions, such as hydrogen bonds, salt bridges, or van der Waals forces, are suggested based on proximity but not confirmed through measurements. Especially weaker, non-classical interactions involving hydrogen atoms are often disregarded, leading to potential misinterpretations of the binding.
4. The dynamic behavior of ligand-protein complexes is not elucidated.
  X-ray crystallography captures a single, static snapshot of a ligand bound to a protein, providing valuable structural information but failing to reveal the dynamic nature of the interaction. Proteins and ligands are inherently flexible and the subtle interplay between enthalpy and entropy is critical for binding affinity and specificity. Crystallography also cannot capture the existence of multiple bound states that often occur in solution, missing key details about the full range of protein-ligand interactions that influence drug efficacy and binding kinetics.
5. ~20% of protein-bound waters are not X-ray observable^7^.
  Water molecules play a critical role in mediating protein-ligand interactions by bridging between the ligand and protein side chains thereby stabilizing binding or modulating the conformational state of the protein. However, in X-ray crystallography, approximately 20% of these bound waters are invisible because they either lack sufficient electron density due to their high mobility or occupy poorly ordered regions that do not diffract X-rays effectively. These undetected waters, especially in flexible or surface-exposed regions, can be crucial to understanding the thermodynamics of binding and hydration networks.
6. X-ray crystallography is “blind” to hydrogen information and complementary techniques are therefore highly important to elucidate H-bonding interactions^8,9^.
  X-ray crystallography relies on electron density to determine atomic positions, but since hydrogen atoms do not possess significant electron density, they are essentially invisible in most X-ray structures. This creates a significant limitation, as hydrogen atoms are fundamental to key interactions like hydrogen bonds. Without directly observing hydrogen atoms, the precise geometry of hydrogen bonds and the protonation states of ionizable groups often cannot be determined accurately, potentially leading to incorrect interpretations of binding interactions. Complementary techniques, such as nuclear magnetic resonance (NMR) spectroscopy, neutron diffraction, or computational methods, are essential for providing detailed information on hydrogen atom positions, hydrogen bonding networks, and the protonation states of residues.
7. Finally, enthalpy-entropy compensation is a fundamental and inevitable problem in rational drug design (subtle interplay between conformational entropy and differential hydration)^10–12^.

Optimizing binding affinity often involves a trade-off between enthalpy (ΔH) and entropy (ΔS), a phenomenon known as enthalpy-entropy compensation. While favorable enthalpic contributions, such as hydrogen bonds or van der Waals interactions, improve binding affinity, they often come at the cost of a decrease in conformational entropy. This occurs because the ligand and protein may adopt more rigid conformations upon binding, leading to a loss of flexibility that negatively impacts the entropy. Additionally, water molecules displaced from the binding site can either release or absorb energy, depending on their arrangement before and after binding, which further complicates this balance. Differential hydration, which refers to the variable behavior of water molecules around the ligand and protein, also plays a key role in this compensation effect. The reorganization of water networks can either enhance or diminish the binding free energy, making it difficult to predict how modifications to the ligand or protein will affect the overall binding process.

While X-ray crystallography remains a cornerstone of structural biology, its inherent limitations, such as challenges with crystallization, poor representation of dynamic protein-ligand interactions, and inability to resolve hydrogen atom details, highlight the need for complementary approaches. NMR spectroscopy provides a powerful alternative by capturing detailed, solution-state interactions and dynamic conformational changes. Table 1 highlights strengths and limitations the 3 main biophysical techniques for structure determination in a drug discovery context.

**Table 1:** Strengths and Limitations of Structure Elucidating Methods

Methods	MW Limit	Resolution	Conformational Dynamics	High-throughput Viable	Hydrogen Information
X-ray Crystallography	No	High resolution ( ~ 1 Å)	No	Yes	No
NMR Spectroscopy	>80 kDa	High resolution ( ~ 1-2 Å)	Yes	Yes	Yes
Cryo-EM	<50 kDa	Medium-high resolution ( ~ 2-5 Å)	Yes	No	Yes

Strengths and Limitations of the three main structure elucidating methods employed in drug discovery based on the number of structures deposited to the Protein Data Bank (RCSB.org)^83^. As of writing of this manuscript the number of protein structures solved via different biophysical techniques were X-ray: 188k, EM: 24k, Solution NMR: 12.7k, Electron-Crystallography: 239, Neutron Diffraction: 224, Solid State NMR: 180.

**Fig. 1:**
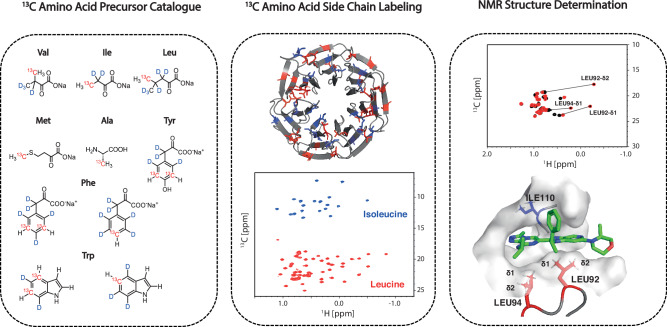
Outline of NMR-SBDD. Starting from ^13^C-labeled amino acids or precursors (left) the protein of interest can be selectively labeled for NMR spectroscopy. X-ray structure (PDB ID 4QL1) and ^1^H-^13^C HSQC spectrum of selective leucine and isoleucine labeled WDR5 (34 kDa) highlights the benefits of this methodology; significantly simplified NMR spectra also amenable for larger protein targets usually outside the range of conventional NMR methods (middle). Substantial chemical shift changes of protein signals induced by aromatic ligand ring-systems can be directly incorporated into 3D structural calculations of protein-ligand complexes. Shown is the example of a ligand interacting with selectively leucine labeled BRD4-BD1 (PDB ID 6XV3)(right)^22^.

In this work, we discuss a novel research strategy termed NMR-Driven Structure-Based Drug Design (NMR-SBDD) which combines a catalogue of ^13^C amino acid precursors, ^13^C side chain protein labeling strategies, and straightforward NMR spectroscopic approaches in combination with advanced computational tools (Fig. 1).

### Advantages of NMR-SBDD

NMR spectroscopy has become an indispensable tool in structure-based drug design, especially in the context of fragment-based drug design (FBDD), as emphasized by numerous key studies^1,13–18^. NMR offers direct access to atomistic information which can help in identifying non-covalent interactions in protein-ligand systems that favorably contribute to the enthalpic component of the binding free energy^19^. In this context, the information encoded in the ^1^H chemical shift is especially relevant since it directly reports on the nature of hydrogen-bonding a proton is potentially involved in. Protons with large ^1^H downfield chemical shift values (higher ppm values) are usually the hydrogen bond donor in a classical H-bond interaction^20,21^, whereas those with a large ^1^H upfield chemical shift value (lower ppm value) correspond to hydrogen bond donors with aromatic ring systems (e.g. in CH-*π* and Methyl-*π* interactions)^22,23^. Both types of interactions have been subject to a plethora of computational studies^24–26^. Having a reliable gauge on the individual energetic contributions of the different types of non-covalent interactions is of significant importance in drug design and has been put forward as a crucial determinant distinguishing best in class from first in class compounds^27^.

Another advantage of NMR is its relatively straightforward sample preparation process. Statistics taken from a Human Proteome Structural Genomics pilot project show that of the proteins that were successfully cloned, expressed and purified only 25% gave rise to crystals suitable for X-ray crystallography^6^. While soaking systems provide high-throughput access to X-ray structures, they are often challenging to establish leaving the rapid generation of 3D protein-ligand structures out of reach for most drug discovery projects. In contrast, NMR is not dependent on the generation of crystals but offers access to protein-ligand structures in solution and can in certain cases assist in the generation of reliable structural ensembles that closely resemble the native state distribution of protein-ligand complexes in solution^28–31^. This is especially important for studying systems with partial disorder, such as linker regions connecting structured domains or intrinsically disordered regions within proteins. These flexible regions often hinder crystallization due to their dynamic nature, making them challenging targets for traditional X-ray crystallography. A detailed discussion of targeting intrinsically disordered proteins (IDPs) and highly dynamic biomolecular condensates can be found elsewhere^32,33^. Moreover, NMR can expand the number of structures possible where certain compounds failed to yield X-ray structures for technical reasons (i.e. poorly diffracting crystal structures)^22^. In addition NMR can provide access to site-specific information about intermolecular interactions, structural dynamics and hydration phenomena in solution. Importantly, historical bottlenecks and shortcomings of the methodology (e.g. limited sensitivity, molecular weight limitations, time consuming and thus expensive signal assignment) are being constantly improved due to continuous advancements in NMR hardware and methodologies (e.g. long lived coherences^34^ and dynamic nuclear polarization^35^) as well as the integration of artificial intelligence (AI) into NMR workflows^36,37^.

Despite technical advancements, challenges related to molecular weight persist, particularly for proteins or complexes exceeding ~50 kDa, where spectral overlap and reduced sensitivity can complicate data acquisition and interpretation. Recent work on TROSY-based experiments in combination with deep learning methods have further extended the molecular weight range accessible to NMR^38^. Alternatively, integrating complementary techniques such as cryo-EM and solid-state NMR data can provide additional structural insights that overcome limitations of individual methods. For example, cryo-EM offers medium-resolution structural data suitable for mapping larger complexes, while NMR provides local atomic-level details and dynamic information. Large proteins have been resolved to near-atomic resolution by combining EM maps with NMR-derived secondary structures and distance restraints underscoring the power of multi-modal strategies in studying high-molecular-weight targets^39^.

In the following sections, we explore how technical challenges, such as limited protein solubility, sensitivity constraints at low protein concentrations, and the traditionally time-intensive process of NMR signal assignment, are effectively addressed by leveraging isotope-labeling strategies in combination with fast and efficient NMR experiments.

### ^13^C Amino Acid Precursor Catalogue

Protein NMR has relied largely on uniform ^15^N labeling of protein backbone nitrogens for detection. While this is useful to reliably detect binding events to the protein of interest, it is not capable of providing site specific information in the absence of laborious amino acid assignment experiments. Additionally, ligand interactions with backbone-amides are less frequent than interactions directly with the respective amino acid side chains. Therefore, the labeling of amino acid side chains with isolated ^1^H-^13^C spin pairs allows site specific information through chemical shift perturbation (CSP) monitoring directly at the protein-ligand interface thereby increases sensitivity in addition to extending the protein molecular weight range. The discovery of a robust and cost-effective amino acid side chain labeling strategy using amino acid precursors in E-coli^40,41^ and the expansion to include suitably labeled aliphatic aromatic amino acid side chains^42–45^ has been instrumental in enabling the NMR detection of isolated ^1^H-^13^C spin pairs. A catalogue of different isotopologues is now easily available via efficient multistep organic synthesis and allow for unique and flexible isotope-labeling patterns in proteins enabling sensitive NMR detection schemes.

### Exploiting ^1^H Chemical Shift Information to guide drug discovery

#### **a)** Protein-detected ^1^H Information

Site-specific information about molecular interactions at atomic resolution can be extracted and used to guide drug-development processes^13^. We have recently shown that CH-π interactions can be very effectively probed by simply monitoring and quantifying CSPs of isolated ^1^H-^13^C spin pair resonances induced by ligand binding^23^. Significant chemical shift changes are induced by CH-π interactions involving aromatic spin systems of both the ligand or the protein and can be directly related to geometric (orientational) parameters^23^. Protons in spatial proximity to an aromatic ring system experience differential effects from strong shielding to weak deshielding, depending on the orientation of the aromatic ring relative to the protons being affected^46^. This information has already been successfully used in the past to orient ligands in the binding pocket^47^. We recently extended our initial work to also cover Methyl-π interactions and show that the extracted NMR information can be used to position ligands in the binding pocket, even when the binding affinity is low^22^.

#### **b)** Ligand-detected ^1^H Information

Chemical Shift information of a ligand in the protein-bound state contains a wealth of information that is rarely used in drug design. An atomistic experimental read-out directly at the protein-ligand interface is valuable for computational chemists to verify their structural model based on solution data^48^. Once an accurate structural model is generated, quantum mechanical methods allow for a decomposition of the binding energy terms of individual atoms engaged in non-covalent interactions with the protein opening the way for a more informed rational design approach.

### Hydration Waters with NMR

Finally, NMR has been demonstrated to also provide access to hydration water in protein systems^49,50^. Again, due to advancements in hardware and pulse sequence development techniques are available to probe the existence of water molecules in protein hydration shells and characterize them with respect to lifetimes and hydrogen bonding strengths. Most importantly, NMR also detects hydration water molecules characterized by a wide range of lifetimes^7,51,52^. In a previous study we have shown that NMR (employing Nuclear Overhauser Effect (NOE) measurements) can be used to identify loosely bound hydration water molecules that can be replaced by established water mimicking chemical moieties and thereby boosting ligand affinity^53^. However, significant progress is still needed to fully capture the diverse timescales of water interactions with biomolecules and harness their potential for drug discovery programs.

### Dynamic SBDD through NMR

An adequate and accurate description of the structural ensemble of a protein complex is crucial in the medicinal chemistry optimization process. NMR spectroscopy has provided ample and undisputable evidence that proteins are not static molecules but populate a range of structural states that dynamically interconvert on a multitude of timescales^54–58^. These dynamic modes comprise local backbone fluctuations, side chain flips (i.e. aromatic rings), relative changes of domain orientations and even changes in oligomeric subunits. Particularly, due to the recent development of NMR techniques with exquisite sensitivity to motional processes even transiently and only sparsely populated conformational substates can be probed and characterized^59,60^. These techniques are well established and can also be reliably used to determine the presence of multiple ligand bound-states^61^. and investigate binding mechanisms (conformational selection *vs* induced fit)^62–64^.

Although, studies of protein-ligand binding processes have already been reported by academic research groups^23,65^ and applications in drug design programs (both in academia and industry) are becoming more popular we feel that the enormous potential of NMR is still not fully exploited. Given the robustness of these techniques and their potential for automation, together with the availability of selectively labeled protein material (see above for amino acid precursor labeling) we thus propose to implement these techniques in the standard repertoire of experimental drug design programs. Arguably the most import reason for applying NMR to SBDD is because it can probe molecular motions can be probed by NMR with atomic resolution giving valuable insight into the structural dynamics of protein-ligand complexes^54–58^.

### A New Drug Design Paradigm

**Fig. 2:**
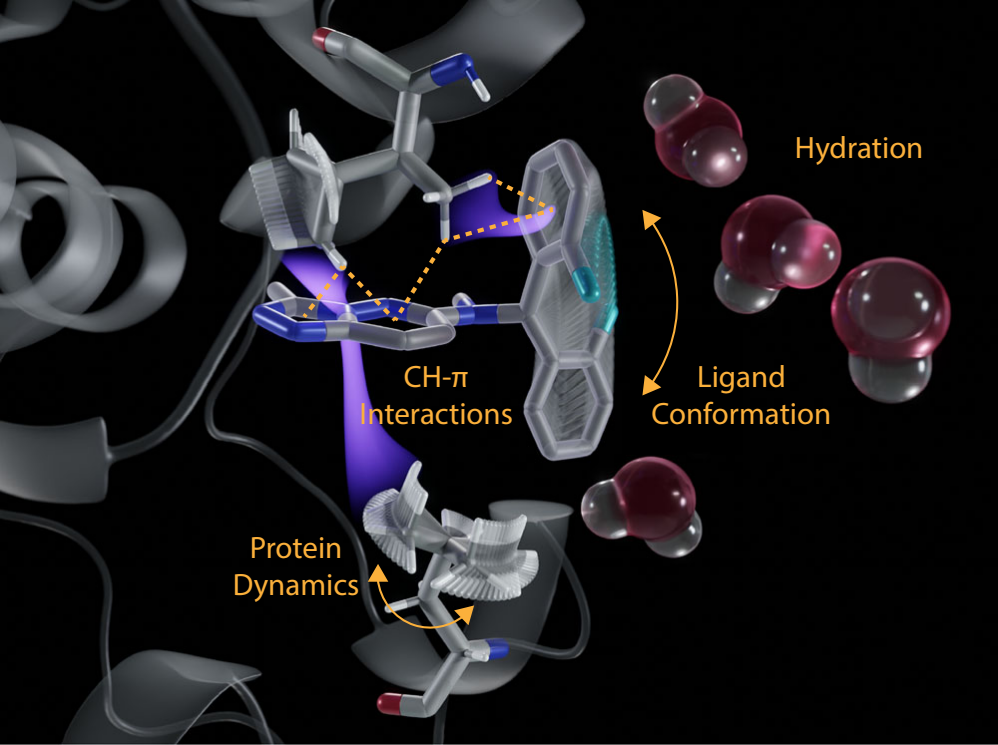
Fundamental Challenges and Deliverables in Drug Design. The four fundamental questions and deliverables required for successful drug design programs are illustrated for a typical protein-ligand complex. **a** The protein-bound conformation of the ligand is necessary (although not sufficient) for a deeper understanding of binding affinities; **b** Disentanglement of protein-ligand interactions across the interface is essential to dissect the energetics of protein-ligand complexes; (interactions of ligand aromatic ring-systems with protein hydrogens are highlighted). **c** Conformational dynamics prevails even in high affinity biological complexes and thereby contributes to conformational entropy; **d** Water molecules are integral parts of protein-ligand complexes and contribute to the binding energies.

In view of the above mentioned advantages of NMR-SBDD we propose a paradigm shift and new design principles, which are addressing the four central problems in drug design: (1) protein exists as conformational ensembles; (2) direct probing (of hydrogen) and subsequent (rational) optimization of individual interactions rather than indirect extrapolations from static structures; (3) residual molecular dynamics in the ligand-bound state contribute to the entropy of binding and is thus a rich source of information in the design process; (4) water molecules are an integral part of the protein-ligand interface and contribute to the binding energetics (Fig. 2).

A single protein-ligand structure is unlikely to adequately represent the actual ensemble of structures present in the solution state, especially in the earlier stages of compound optimization. Therefore, ensembles of protein-ligand structures (“movies”) representative of the protein-ligand conformational space should be used for drug design rather than static “photos”^66^. In order to provide accurate structural ensembles of the protein we propose to use a sufficiently large structural ensemble stemming from, for example, molecular dynamics (MD) simulations or alternatively, deep learning approaches. Although AlphaFold (AF) was originally designed to predict static structures of proteins, recent applications have demonstrated its capability to assess multiple conformations and/or structural heterogeneity of proteins by reducing the depth of the input multiple sequence alignments (MSAs)^67–69^. These structures can in turn serve as starting points for additional MD simulations further increasing the accessible protein conformational space. Recent advancements in experiment-guided structure prediction have demonstrated that integrating experimental data into deep learning models can significantly improve the accuracy of conformational ensembles, offering a more realistic representation of protein-ligand interactions^70^. Importantly, even cryptic pockets not detectable by extensive MD simulations were identified suggesting that indeed deep learning methods allow detection and further analysis of multiple conformational states^71–73^.

While AF can generate multiple structural conformations, these do not necessarily represent experimentally validated ensemble data. The conformational diversity provided by AF is primarily an outcome of sequence similarity-based structural inference, rather than an explicit representation of intrinsic molecular flexibility observed in solution. To ensure that in-silico generated models represent binding-relevant structures in the solution-state ensemble, we make use of experimentally derived CSP data of the protein ligand complex. Computational docking algorithms are employed to provide possible ligand poses for the individual conformers of the protein and NMR spectroscopic data (CSPs) are in turn used to extract the most probable binding poses from the conformational ensemble by comparing calculated with experimental CSP values. Especially CSPs induced by aromatic ring systems offer an accurate and reliable constraint given their potentially large absolute values and geometrical dependence^23,74^. Ligand ^1^H chemical shifts can also be incorporated using more advanced quantum mechanical (QM) methods^75^ and have been shown to improve the accuracy of solution structures of protein-ligand complexes^48^.

A promising approach for aligning in-silico generated and experimentally derived NMR ensemble data is the use of ensemble reweighting techniques, such as Maximum Entropy (MaxEnt)^76–78^ reweighting to select for appropriate computational structures that are able to reproduce the experimentally derived structural ensemble based on NMR CSP data. Starting from a “prior” distribution of initial conformations, obtained from e.g. MD or Markov Chain Monte Carlo (MCMC) simulations, these methods give an ensemble estimate by minimally reweighting the prior while simultaneously matching the experimental data. Even IDPs can be adequately described using this approach^79,80^ and preliminary data in our laboratory suggest the feasibility to use MaxEnt approaches to reweight conformational ensembles of protein-ligand complexes. The thereby generated ensemble of binding poses can subsequently be used for medicinal chemistry optimization of early hits. Given the huge potential of machine learning methods it remains of critical importance to further grow public-domain experimental data as well as benchmarking initiatives in order to better train the underlying models^81^.

### Outlook

NMR-assisted Structure-Based Drug Design has made significant advances in recent years, but further progress is required to meet the increasingly challenging environment of modern drug discovery. As drug targets become more complex and dynamic, NMR-SBDD’s ability to capture interactions in solution presents a unique opportunity. Below, we outline the key opportunities and challenges for the future development of NMR-SBDD.

#### Opportunities

- **Advances in Hardware and AI-Assisted NMR**: Continuous improvements in NMR hardware, particularly in sensitivity and resolution, open new possibilities for studying larger and more complex protein-ligand systems. Integration of AI and machine learning models into NMR data processing will enhance the generation of precise structural ensembles from limited experimental data, improving integration with computational drug design.
- **Deep Learning for Structural Ensembles**: Deep learning approaches, such as AF, show potential in predicting multiple conformational states of proteins, enabling exploration of cryptic binding pockets and transient binding states. These tools, when combined with NMR, could facilitate the design of allosteric modulators or inhibitors targeting previously inaccessible protein regions.
- **Fragment-Based Drug Discovery**: NMR’s ability to detect weak and transient interactions (e.g., CH-π interactions, hydrogen bonding) provides a unique opportunity to identify and optimize small fragment hits for which protein-ligand structure generation is out of reach for classical approaches.
- **NMR-assisted Water Mapping**: Water molecules play a critical role in modulating protein-ligand interactions. NMR techniques have shown promise in mapping these effects, potentially informing the design of water-mimicking ligands. Combining NMR with cutting edge water-mapping approaches^82^ holds a great promise.
- **Multi-modal Integration**: Combining NMR-SBDD with other structural biology techniques such as X-ray, cryo-EM and computational methods can provide a comprehensive view of protein-ligand interactions.

#### Challenges

- **High-Throughput Limitations**: Achieving high-throughput capabilities in NMR remains a challenge. Streamlined workflows integrating isotope labeling, automated data analysis, and computational modeling are essential for scaling NMR-SBDD.
- **Scalability**: Molecular weight limitations restrict NMR applications for larger proteins and complexes. Advances in isotope-labeling strategies and sensitivity improvements are key to address these limitations.
- **Validation of Computational Models**: Computational models require robust validation, especially for proteins with high structural plasticity or cryptic binding pockets. This is critical to ensure accuracy and reliability.
- **Sample Preparation and Signal Interpretation**: Challenges in isotope labeling strategies and interpreting complex NMR spectra for large protein systems remain a bottleneck, requiring further refinement.
- **Hydration Dynamics**: Capturing hydration effects in protein-ligand systems demands sophisticated experimental setups and protocols that are sensitive yet feasible for high-throughput applications.

By addressing these challenges and leveraging the opportunities, NMR-SBDD can continue to evolve as a powerful tool in drug discovery, providing unique insights into the dynamic nature of protein-ligand interactions.

### Conclusion

NMR-SBDD represents a drug discovery approach that has the potential to unlock many currently undrugged proteins, particularly as we move towards a more dynamic understanding of protein-ligand interactions. The future of drug discovery will likely see a convergence of NMR with AI, deep learning, and other structural biology methods to allow us to drug targets which are beyond our current technical limits. However, challenges remain in scalability, automation, and integration with high-throughput approaches, all of which need to be addressed to unlock the full potential of NMR-SBDD.
